# Supplementary material for: Giant sponge grounds of Central Arctic seamounts are associated with extinct seep life
Source: Nat Commun. 2022 Feb 8;13:638. doi: 10.1038/s41467-022-28129-7 (PMC8826442; doi:10.1038/s41467-022-28129-7)
Supplement: Supplementary file 1 — Supplementary Information [file 41467_2022_28129_MOESM1_ESM.pdf]

Supplementary Information for

Giant sponge grounds of Central Arctic seamounts are associated with extinct seep life

T. M. Morganti, B. M. Slaby, A. De Kluijver, K. Busch, U. Hentschel, J. J. Middelburg, H. Grotheer, G. Mollenhauer, J. Dannheim, H. T. Rapp, A. Purser, A. Boetius

Correspondence to: tmorgant@mpi-bremen.de and antje.boetius@awi.de

This PDF file includes:

Supplementary Methods
Supplementary Figures 1 to 5
Supplementary Tables 1 to 3

Other Supplementary Information for this manuscript include the following:

Supplementary Data 1 to 3

Supplementary Methods

OFOBS mapping and counts of individuals

The peaks of the Langseth Ridge were rather heterogeneous in slope angle and facing aspect, with occasional outcrops of underlying rock piercing through the other habitat categories, apparently influencing the proportion of sponge, spicule and worm tube coverages. This physical topographical variability across scales of meters was observed throughout all OFOBS deployments, resulting in more pronounced localized variations in sponge abundances than might be indicated from the depth parameter alone. Sponge abundances on the flat seamount peaks (Fig. 2D) were observed to be on average 6 ind. m^{-2} (n=54 images, SD=1.7, the d sponge coverage category). Sponge abundances on the shallow sloping upper flanks of the seamounts (Fig. 2C) were on average 2.8 ind. m^{-2} (n=211 images, SD=1.1, the c sponge coverage category). A peak density of 11 ind. m^{-2} was observed in the saddle at depths of down to 1000 m between the two southerly seamount peaks. The sponge density was lower on the surrounding seafloor, as the steeper flank edge was approached, at 0.8 ind. m^{-2} (n=361 images, SD=0.7, the b sponge coverage category) (Fig. 2B), with the steeper walls and occasional rocky outcrops /sudden steps only supporting occasional sponge individuals and communities of a few 10s of sponges (Fig. 2A).

Preparation for stable isotope analyses

Sponges were cleaned from epibionts and dissected into small pieces. At least three subsamples for each specimen were analyzed, in any sample the sponge cortex was not included. For small sample fauna, as crustaceans, hydrozoans and polychaetes, entire individuals were analyzed or pooled together with additional individuals to reach the minimum required mass for isotopic analysis. Samples were subsequently lyophilized for 48hr in a Scala Scientific TFD5503, ground to a fine powder using an agate and mortar pestle, prior to loading into Ag capsules. Samples were processed following the method described in [1]. Briefly, biological samples were acidified by adding 10µl of 5% HCl repeatedly until reactions ceased to remove inorganic carbon. Samples were then dried at 50°C for at least 1hr under a fume extractor. Sediment samples were acidified with 30% HCl and then heated to 120°C for at least 1hr. GF/F filters were put in a 37% HCl vapor bath for 30 minutes and then dried at 60°C for at least 12hr. The Ag capsules were pinched closed and stored in a desiccator until analysis. Samples were combusted at high temperature (1020°C), separated chromatographically and then analyzed using an Elemental Analyzer (Flash IRMS, Thermo EA) coupled with Isotope Ratio Mass Spectrometer DeltaV, Thermo (EA IsoLink™ IRMS System). Quality control standards from the in-house reference materials nicotine amide (N and C), ammonium sulfate (N), and graphite quartzite (GQ) for C

calibrated against IAEA N1 and IAEA N2 reference material were run for each isotope and for each set of samples. Isotope values are expressed in δ (‰) notation, which is the per mil deviation of a sample (R_{sample}, $^{13}C/^{12}C$, $^{15}N/^{14}N$) relative to the isotope ratio of a standard material ($R_{standard}$) (Vienna Pee Dee Belemnite) and atmospheric N_2 as international standards for carbon and nitrogen respectively, and calculated as following:

$$\delta X=[(R_{sample}/R_{standard})-1] \times 1000$$

X=^{13}C or ^{15}N and R is the ratio of the heavy to light isotope ($^{13}C/^{12}C$, $^{15}N/^{14}N$).

Stable isotope statistical analyses

Bulk sponge isotopic values ($\delta^{13}C$ and $\delta^{15}N$) were analyzed using a linear mixed model effect with sponge species as the fixed factor (level 4: *G. parva*, *G. hentscheli*, *S. rhaphidiophora* and *G. parva* juveniles) and sampling sites as the random factor (level 4: NM, CM, KM and CS) in order to test for differences between sponge species controlled by the variability of the random factor. A second linear mixed model was run to test differences in $\delta^{13}C$ and $\delta^{15}N$ values between those measured from sponges and from other groups of sampled fauna, taking into consideration the location of sample collection (as test random factor). The macrofauna samples (bryozoans, hydrozoans, crustaceans and asteroids) were grouped together as 'macrofauna samples' for this test. Only one calcareous sponge was collected for analysis, and therefore eliminated due to low limited sample size (n=1). Tukey Post Hoc tests were run to test the differences between the mean of all groups (unplanned pairwise comparisons) controlling for Type 1 error [2].

For bivariate isotopic niche space identification, the data were grouped by sample type. Isotopic niche area of each sample type was estimated by calculation of the standard ellipse area (SEAc) using stable isotope Bayesian Ellipses [3]. Since the required absolute minimum sample size is 3 in order for the various ellipses and corresponding metrics to be calculated, for crustacean, starfishes, sea-ice matter and calcareous sponge the ellipses were not calculated due to limited sample size (n<3). Alternatively, the method calculated the SEAc after application of a small sample size correction with ellipses estimated enveloping 95% of the data. Stable isotope analysis (SIAR) was also run in order to estimate the relative proportion of different food sources to the consumer diet [4]. The model was run using ^{13}C and ^{15}N stable isotope values from the putative main sources: suspended POM and siboglinid tubes from this study, and DOM and DIC from the literature [5–8].The model was run using two different trophic transfer fractionation factors: 0.5 ± 0.5 ‰ for ^{13}C and 3.5 ± 0.5% for ^{15}N for metazoan food web and 0.5 ± 0.5 ‰ ^{13}C and 1.5 ± 0.5% ^{15}N considering a substantial microbial contribution to the holobiont metabolism [9,10].

The linear mixed models and isotopic niche area analyses were performed using package lme4 v.1.1-21 and SIBER v.2.1.4 [Stable Isotope Bayesian Ellipses] respectively in R (version 3.6.1) and plots were generated with R studio.

Radiocarbon dating $\Delta^{14}C$

Radiocarbon analyses were carried out at the MICADAS (Mini Carbon Dating System; [11]) facility at the Alfred-Wegener-Institute in Bremerhaven, Germany according to published standard operation procedures [12]. Sponge tissue samples were freeze-dried, sub samples were cut out using a scalpel, visible spicules were removed, and material was finely ground. The bulk organic matter samples were acidified in order to remove inorganic carbon by using HCl and subsequently combusted using an Elemental analyzer. The bivalve shell was pre-cleaned with HCl. Carbonate samples were hydrolyzed with phosphoric acid using the carbonate handling system (CHS). Depending on sample size, the measurements were made as graphite target (> 200 µg C) following graphitization in an AGE system [13] or CO_2 gas (20-100 µg C) directly introduced into the MICADAS ion source via a Gas Interface System [14]. Results were reported as conventional radiocarbon ages [15]. These notations involve a correction for isotope fractionation occurring during sample formation or processing; *i.e.*, all data were normalized to a $\delta^{13}C$ value of −25‰. In this way, carbonate and organic matter samples can be compared. Conventional radiocarbon ages were not converted to calendar age because information on reservoir age is not available, and because we were only interested in the relative age distance between the samples. One subsample from the large sponge specimen was found to contain ^{14}C contamination ($F^{14}C$ value >> 1) from an unknown source, and therefore was excluded from further analysis.

For the $\Delta^{14}C$ value of PLFAs, specific compounds of interest (bacteria and sponge specific PLFAs) were isolated using established procedures via preparative capillary gas chromatography on an Agilent 7890B connected to a preparative fraction collector (PFC, Gerstel) [16,17]. The isolated compounds were subjected to purity check (< 1% impurity) on the GC-FID and subsequently dissolved in DCM, transferred in 25µL liquid tin capsules and placed at 40°C until DCM evaporated. The measurements were made by combustion in the EA directly introduced into the MICADAS ion source via a Gas Interface System (GIS, Ionplus [18]. Details of step-by-step protocol for PLFAs $^{14}\Delta C$ analysis are detailed in [12].

Amplicon data analyses

DNA was extracted from ~0.25 g sponge, or 50% of a filter for the seawater sample analyses, using the DNeasy Power Soil Kit (Qiagen, Cat. No. 12888-100). Quantity and quality of the DNA extracts was assessed by NanoDrop spectrophotometry and PCR with universal 16S primers and subsequent gel electrophoresis. The V3V4 variable regions of the 16S rRNA gene were

amplified with the primer pair 341F-806R (5'-CCTACGGGAGGCAGCAG-3' [19] and 5'-GGACTACHVGGGTWTCTAAT-3' [20]). PCR products were normalized, pooled, and DNA libraries sequenced using v3 chemistry on a MiSeq platform (MiSeqFGx, Illumina) at the Institute of Clinical Molecular Biology (IKMB) of Kiel University, allowing zero mismatches in the barcode sequence for demultiplexing. The Qiime2 environment (version 2018.11; [21]) was used to process raw reads. Exact Amplicon Sequence Variants (ASVs) were generated with the help of the DADA2 algorithm [22]. Based on ASVs, phylogenetic trees were calculated using the FastTree2 plugin. Based on a primer-specific Naive Bayes taxonomic classifier, trained on the Silva 138 99 % OTUs 16S database, taxonomic classification of representative ASVs was achieved.

SUPPLEMENTARY FIGURES

Supplementary Figure 1

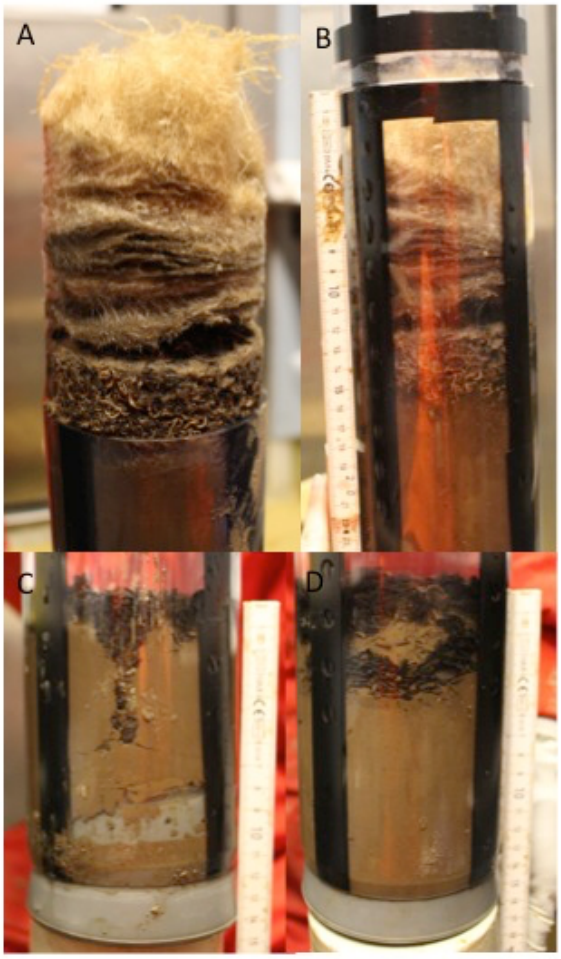

Supplementary Figure 1. Views of different substrata from the multi-corer samples. Tubes are 10 cm in diameter. **(A)** Typical spicule-tube mat like structure that extends up to 15 cm; **(B)** chitin and proteinaceous tube layer with relatively poor spicule matrix. Tubes identified as *Polybrachia* sp. by HT Rapp and A Vedenin. Photo credits Jakob Barz.

Supplementary Figure 2

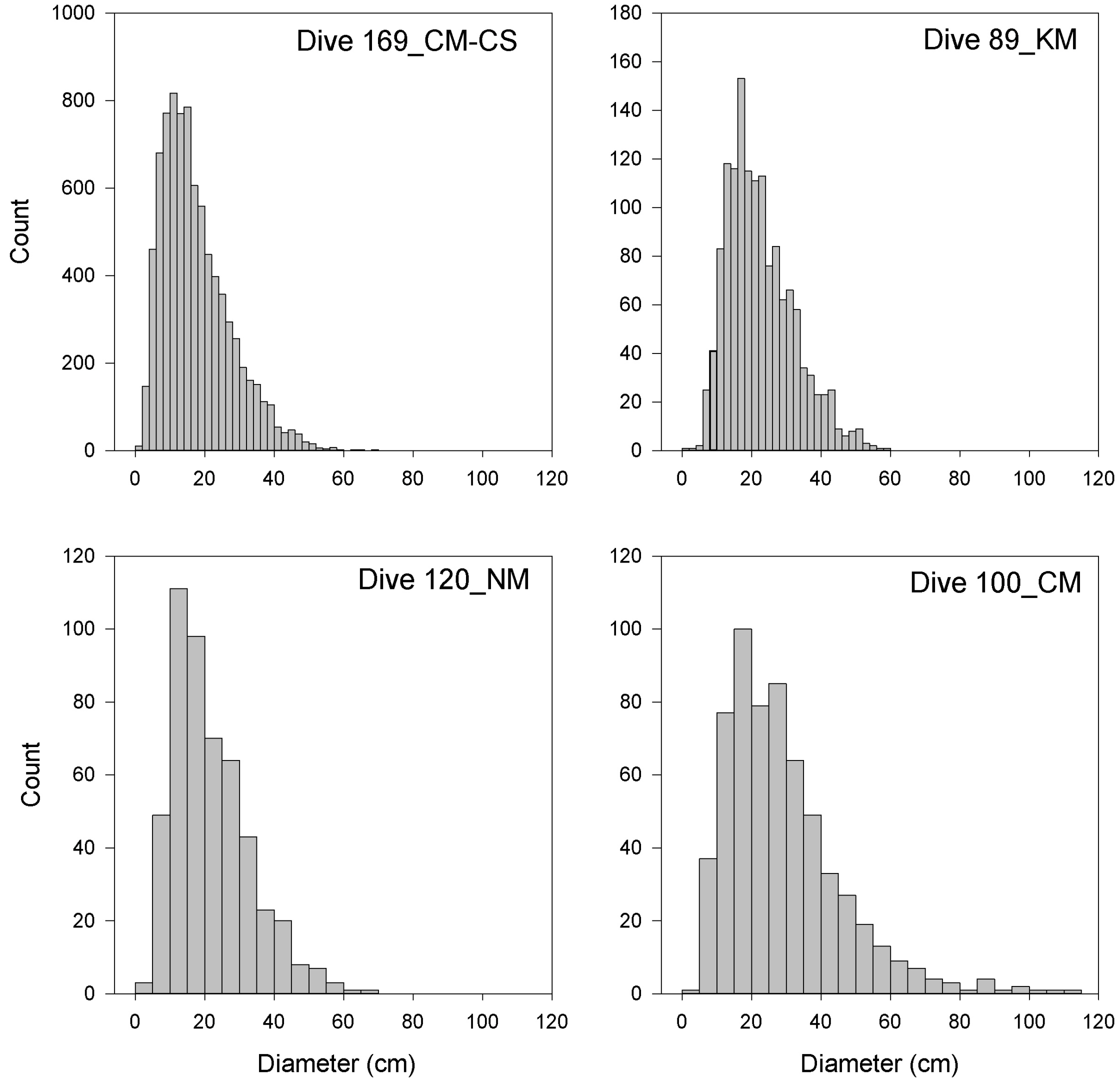

Supplementary Figure 2. Size distribution analysis of sponge specimens (diameter ≥1 cm) over the three summits and saddle. CM-CS: Central Mount and Central Mount saddle; NM: Northern Mount; KM: Karasik Seamount; CM: Central Mount.

Supplementary Figure 3

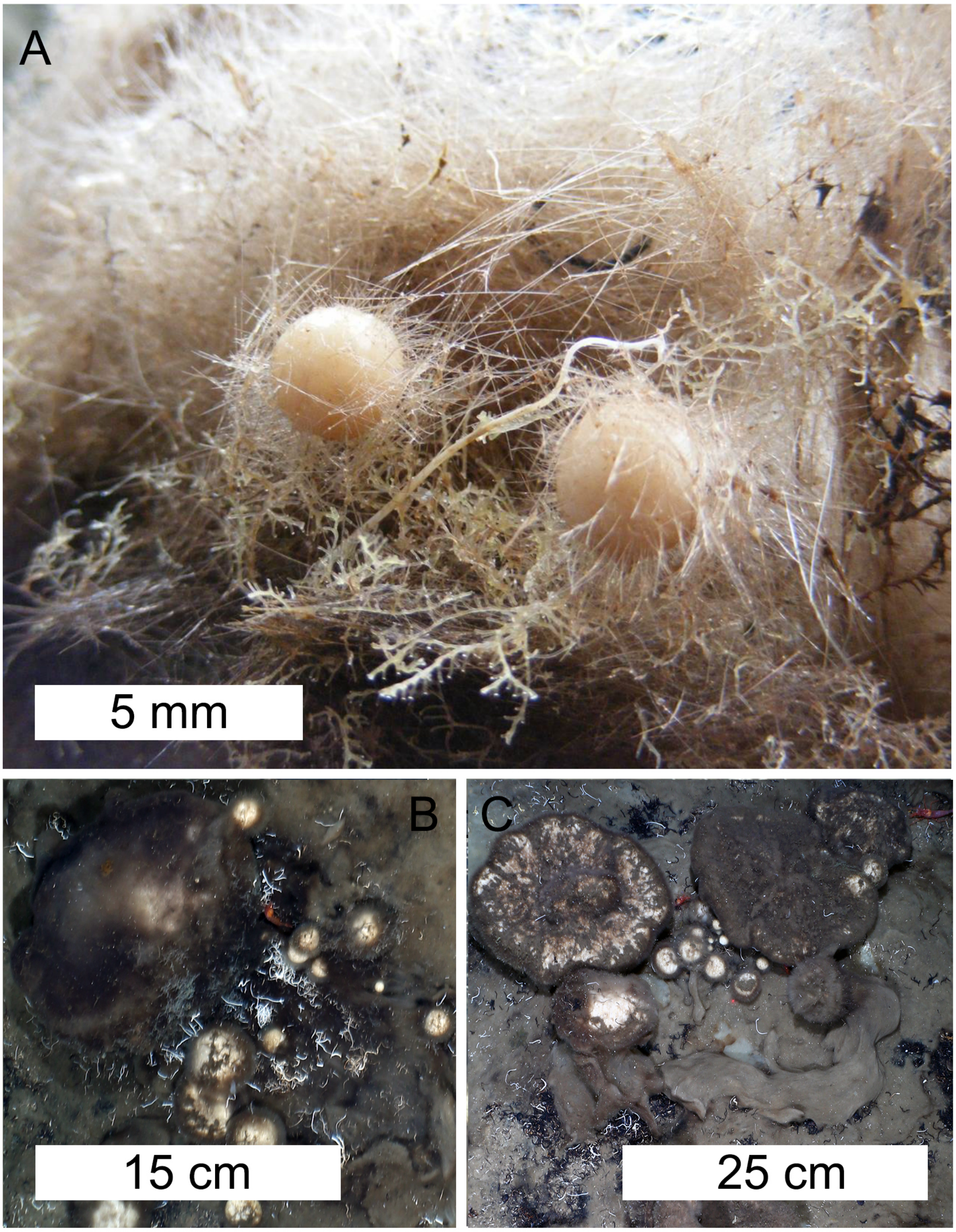

Supplementary Figure 3. (A) Sponge juveniles within the sponge spicules on a box core sponge sample (photo credits A. Boetius); **(B)** juveniles in proximity to adults *in situ*, observed during OFOBS PS101_100 (CM); (C) juveniles in between two large sponges, observed during OFOBS PS101_169 (CM) (photo credits PS101 AWI OFOBS system).

Supplementary Figure 4

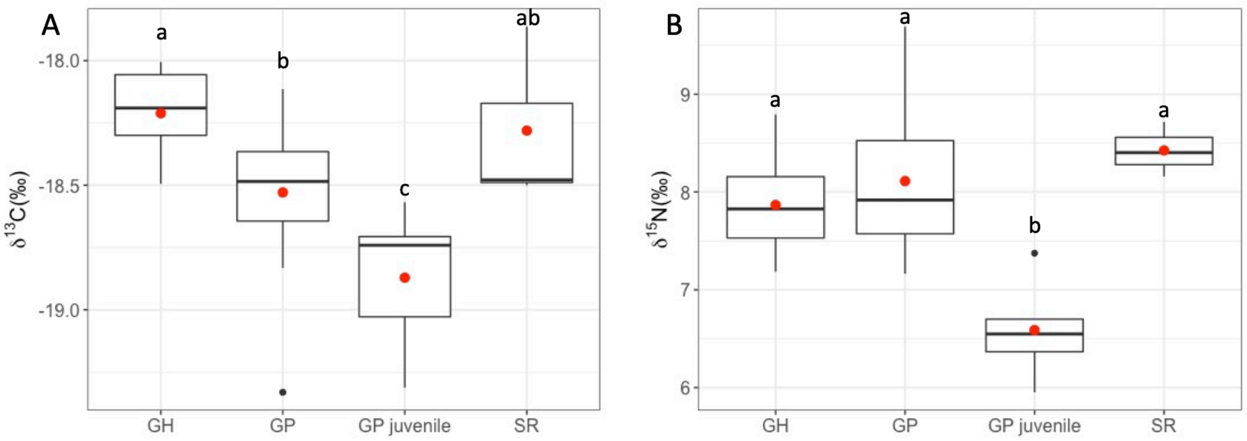

Supplementary Figure 4. Bulk δ^{13}C (A) and δ^{15}N (B) values (‰) of the analyzed species: GP= *G. parva* (*n*=15); GH= *G. hentscheli* (*n*=8); SR= *S. rhaphidiophora* (*n*=3); GP juvenile= *G. parva* juveniles. The letters (a,b,c) indicate significant differences ($p<0.01$) tested with post-Hoc Tukey test between the three species and the GP juveniles. The mean and median are represented by the red dot and black line on each boxplot, respectively. The lower and upper hinges of the boxes correspond to the 25th and 75th percentiles and the whiskers represent the 1.5 x inter-quartile range (IQR) extending from the hinges. The black dots outside the boxplots represent outliers.

Supplementary Figure 5

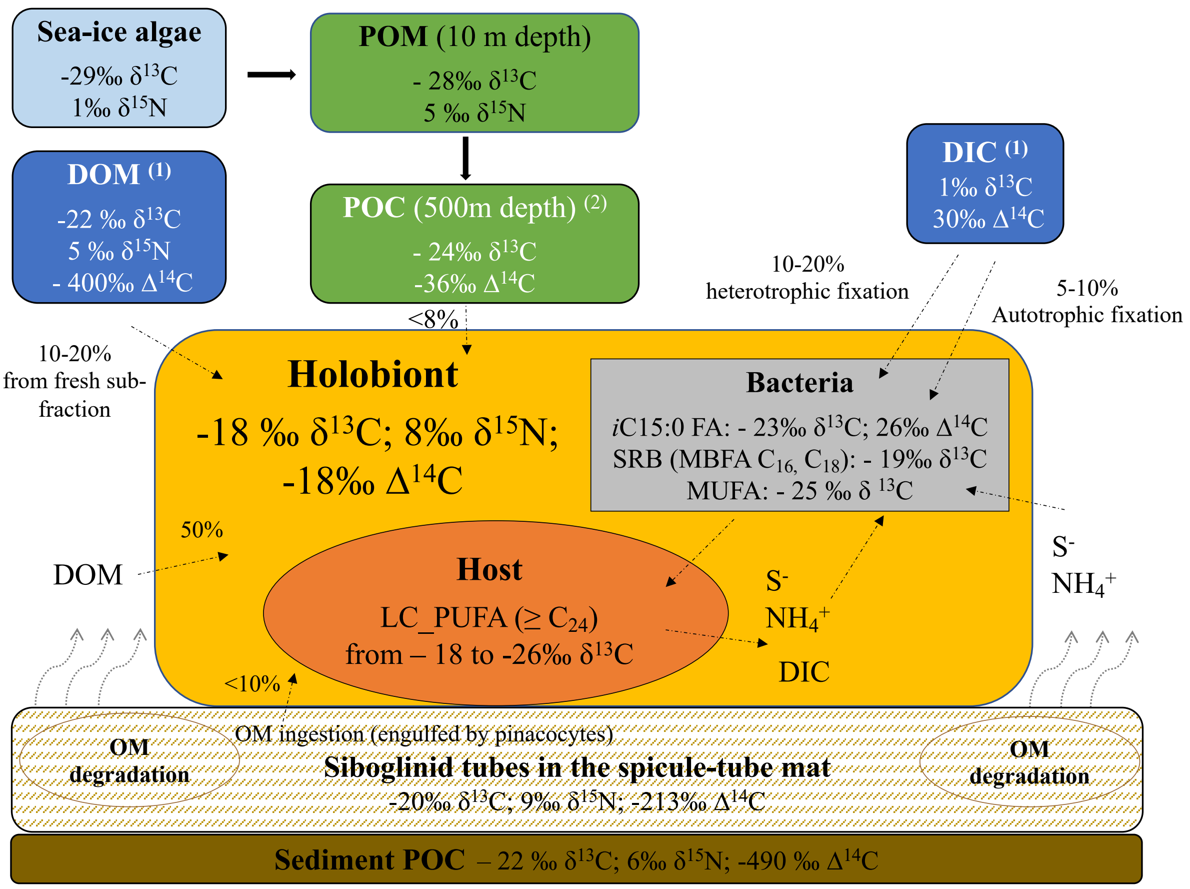

Supplementary Figure 5 Conceptual diagram summarizing the different food and energy sources and their relative contribution to the sponge diet.

POM= Particulate organic matter; POC= Particulate organic carbon; DOM= Dissolved organic matter; DIC= Dissolved inorganic carbon; FA= Fatty acid; SRB=Sulphur reducing bacteria; MBFA=Methyl branched fatty acid; MUFA: Monounsaturated FA.

(1) Druffel, E. R. M., S. Griffin, C. S. Glynn, R. Benner, and B. D. Walker (2017). Radiocarbon in dissolved organic and inorganic carbon of the Arctic Ocean,Geophys. Res. Lett., 44, 2369–2376, doi:10.1002/2016GL072138.
(2) Griffith, D. R., McNichol, A. P., Xu, L., McLaughlin, F. A., Macdonald, R. W., Brown, K. A., and Eglinton, T. I.: Carbon dynamics in the western Arctic Ocean: insights from full-depth carbon isotope profiles of DIC, DOC, and POC, Biogeosciences, 9, 1217–1224, https://doi.org/10.5194/bg-9-1217-2012, 2012.

SUPPLEMENTARY TABLES

Supplementary Table 1. List of identified bivalves at the Langseth Ridge. Latitude (Lat), longitude (long) and sample depth (depth, m) are provided in the table. All samples were collected using box corer.
NM: Northern Mount; CM: Central Mount; CS: Central Mount saddle; KM: Karasik Seamount.

Station	Summit	Lat	Long	Depth (m)	Species
PS101/198	NM	86°51.50'N	61°32.81'E	679	*Limatula hyperborea, Portlandia arctica*
PS101/200	NM	86°51.49'N	61°35.76'E	684	*Limatula hyperborea*
PS101/197	NM	86°51.37'N	61°34.72'E	648	*Limatula hyperborea*
PS101/154	CM	86°49.54'N	61°50.32'E	795	*Limatula hyperborea, Bathyarca frielei, Portlandia arctica, Boreacola maltzani*
PS101/155	CM	86°49.35'N	61°43.69'E	796	*Limatula hyperborea, Mya* sp.
PS101/206	CM	86°49.46'N	61°57.99'E	1011	*Limatula hyperborea, Portlandia arctica, Bathyarca frielei*
PS101/190	CS	86°46.27'N	61°30.63'E	1156	*Bathyarca frielei*
PS101/208	CS	86°46.99'N	61°39.73'E	1122	*Policordia jeffreysi, Portlandia cf. arctica, Portlandia* sp.*, Limatula hyperborea, Portlandia arctica, Cuspidaria* sp.*, Cetoconcha* sp.*, Yoldiella cf. frigida, Cyclopecten* sp.
PS101/92	KM	86°42.61'N	61°19.56'E	663	*Limatula hyperborea, Boreacola maltzani, Portlandia cf. arctica. Astarte* sp.
PS101/93	KM	86°42.54'N	61°19.80'E	622	*Limatula hyperborea, Lyonsia arenosa, Portlandia cf. arctica*
PS101/94	KM	86°42.54'N	61°20.40'E	700	*Limatula hyperborea*

Supplementary Table 2. Results of post-hoc Tukey tests comparing $\delta^{13}C$ (light blue) and $\delta^{15}N$ (light orange) values (‰) between (a) sponge species, and (b) sample types. Statistically significant *p*-values (p<0.05) are in bold. Macrofauna sample type includes hydrozoans, bryozoans, crustaceans and asteroids.

Zoopl. = Zooplankton; POM susp= suspended particulate organic matter; Sed.= Sediment; Poly. tubes= Polychaete tubes; Sibo. Tubes= Siboglinid tubes.

a.

Sponge species	*G. parva*	*G. parva* juveniles	*G. hentscheli*	*S. rhaphidophora*	
G. parva	-	**<0.0001**	0.4	0.8	
G. parva juveniles	**0.009**	-	**<0.0001**	**<0.0001**	**δ^{15}N**
G. hentscheli	**0.007**	**<0.0001**	-	0.4	
S. rhaphidophora	0.280	**0.005**	0.423	-	
		**δ^{13}C**			

b.

Sample type	Fecal pellets	Zoopl.	POM susp	Sea-ice matter	Sed	Poly. tubes	Sibo. tubes	Sponge	Macro-fauna	
Fecal pellets	-	**0.0006**	0.9557	0.2623	0.1397	**<0.0001**	**<0.0001**	**<0.0001**	**<0.0001**	
Zoopl.	0.8045	-	**0.0129**	**<0.0001**	0.0633	**0.0001**	0.2065	0.9953	**<0.0001**	
POM susp	0.9997	0.9697	-	**0.0318**	0.8466	**<0.0001**	**<0.0001**	**<0.0001**	**<0.0001**	
Sea-ice matter	1.00	0.9630	0.9999	-	**0.0010**	**<0.0001**	**<0.0001**	**<0.0001**	**<0.0001**	**δ^{15}N**
Sed.	**0.0040**	0.3054	**0.0091**	0.2195	-	**<0.0001**	**<0.0001**	**<0.0001**	**<0.0001**	
Poly. tubes	1.00	0.9640	1.00	1.00	0.0638	-	**0.0162**	**<0.0001**	0.5683	
Sibo. tubes	**0.0003**	**0.0367**	**0.0007**	0.0549	0.7994	**0.0080**	-	0.1250	0.0792	
Sponge	**<0.0001**	**0.0006**	**<0.0001**	**0.0127**	**0.0238**	**0.0004**	0.9945	-	**<0.0001**	
Macro-fauna	**<0.0001**	**<0.0001**	**<0.0001**	**0.0012**	**0.0001**	**<0.0001**	0.2419	0.2468	-	
					**δ^{13}C**					

Supplementary Table 3. Phospholipid derived fatty acids (PLFAs) relative abundance (%) and respective $\delta^{13}C$ (‰). Data are expressed as mean ± standard deviation.

PLFAs	Biomarker	*Geodia parva* (*n*=9) Relative abundance (%)	*Geodia parva* (*n*=9) $\delta^{13}C$ ‰	*Geodia hentscheli* (*n*=5) Relative abundance (%)	*Geodia hentscheli* (*n*=5) $\delta^{13}C$ ‰	*Stelletta rhaphidophora* (*n*=2) Relative abundance (%)	*Stelletta rhaphidophora* (*n*=2) $\delta^{13}C$ ‰
C16:0	Palmitic Acid	3.8 ± 0.6	-23.4 ± 1.4	4.2 ± 0.6	-23.7 ± 0.6	4.3 ± 0.2	-23.2 ± 0.5
C14:0	General	0.9 ± 0.2	- 23.4 ±	1.3 ± 0.2	-22.8 ± 1.2	1.3 ± 0.4	-22.7 ± 0.3
C15:0	General	0.4 ± 0.2	-23.9 ± 1.2	0.5 ± 0.3	-23.3 ± 1.2	0.5 ± 0.0	-23.3 ± 1.5
C18:0	General	2.3 ± 0.5	-22.7 ± 2.0	3.1 ± 0.4	-21.9 ± 1.2	2.9 ± 0.5	-21.9 ± 0.1
C23:0	General	0.7 ± 0.5	-23.0 ± 2.0	0.2 ± 0.3	-	0.5 ± 0.6	-21.3
*i*C15:0	Bacteria	3.0 ± 0.5	-24.3 ± 1.1	4.5 ± 0.7	-24.1 ± 0.8	5.7 ± 0.4	-23.2 ± 0.2
*ai*C15:0	Bacteria	1.8 ± 0.4	- 21.4 ±	2.9 ± 0.7	-22.3 ± 0.9	4.5 ± 0.6	-22.1 ± 0.1
*i*C17:0	Bacteria	0.7 ± 0.3	-25.6 ± 1.5	0.8 ± 0.1	-25.9 ± 1.2	0.8 ± 0.2	-25.3 ± 0.2
*ai*C17:0	Bacteria	0.9 ± 0.2	-23.3 ± 1.5	1.0 ± 0.2	-23.2 ± 1.1	0.8 ± 0.1	-22.4 ± 0.7
Me-C14:0	Bacteria	0.8 ± 0.3	-25.2 ± 1.4	1.5 ± 0.4	-24.3 ± 1.6	1.8 ± 0.0	-20.3 ± 1.7
Me-C15:0	Bacteria	2.3 ± 0.5	-24.6 ± 1.3	1.7 ± 0.5	-27.2 ± 2.2	1.9 ± 0.9	-24.0 ± 1.1
10-Me-C17:0	Bacteria	1.6 ± 0.2	-24.4 ± 1.0	1.5 ± 0.1	-25.3 ± 0.9	1.5 ± 0.2	-25.0 ± 0.7
8/9/10/11-MeC16:0	Bacteria	9.1 ± 1.3	-20.4 ± 1.2	10.6 ± 0.5	-20.3 ± 0.3	11.2 ± 1.3	-20.2 ± 0.1
9/10/11-MeC18:0	Bacteria	13.0 ± 2.4	-19.3 ± 1.2	16.7 ± 3.3	-18.5 ± 0.7	20.7 ± 0.9	-18.8 ± 0.3
*(a)i*C16:1ω	Bacteria	0.8 ± 0.4	-23.0 ± 1.2	1.1 ± 0.1	-22.0 ± 1.2	1.2 ± 0.3	-19.8 ± 0.9
C16:1ω(5/7/9)	Bacteria	16.8 ± 2.4	-23.0 ± 1.1	18.7 ± 4.0	-23.8 ± 1.1	18.7 ± 0.9	-25.1 ± 2.0
iC17:1ω7	Bacteria	2.0 ± 0.7	-26.4 ± 1.5	3.3 ± 0.9	-26.5 ± 1.6	3.3 ± 0.7	-25.0 ± 0.4
C18:1ω(9/7/9t)	Bacteria	6.3 ± 0.9	-24.5 ± 1.4	6.0 ± 1.2	-24.3 ± 0.6	3.8 ± 0.9	-25.4 ± 0.6
*i*C19:1ω12	Bacteria	1.6 ± 0.4	-18.9 ± 2.6	2.4 ± 0.4	-17.6 ± 1.0	1.7 ± 0.4	-19.8 ± 1.5
Cy-C17:0	Bacteria	-	-	0.3 ± 0.3	-23.2 ± 1.8	0.3 ± 0.4	-24.5 ± 0.0
Cy-C19:0	Bacteria	0.6 ± 0.4	-25.5 ± 1.1	0.9 ± 0.7	-27.6 ± 1.2	1.2 ± 0.2	-25.4 ± 0.7
C20:4ω6/C20:5ω3	Algae	0.4 ± 0.3	-31.8 ± 2.4	0.4 ± 0.4	-30.9 ± 3.4	2.0 ± 0.7	-24.5 ± 0.5
C22:6ω3	Algae	1.3 ± 0.7	-28.7 ± 1.4	0.4 ± 0.2	-29.6 ± 2.0	-	-
C22:2ω	-	1.6 ± 2.4	-18.2 ± 2.1	0.1 ± 0.4	-	2.1 ± 3.0	-18.8 ± 1.5
C24:2ω?	Sponge	1.7 ± 0.5	-21.1 ± 1.1	0.2 ± 0.3	-	-	-
C25:2ω?	Sponge	19.2 ± 7.9	-22.3 ± 0.8	0.9 ± 0.6	-22.8 ± 3.0	-	-
C26:2ω/C26:3ω?	Sponge	0.3 ± 0.4	-	11.7 ± 4.5	-22.0 ± 0.5	-	-
C27:2ω?	Sponge	-	-	1.3 ± 1.2	-21.1 ± 1.0	-	-
C28:2,3ω?	Sponge	1.9 ± 0.8	-24.0 ± 1.1	-	-	-	-
C29:2ω?	Sponge	-	-	-	-	7.9 ± 3.5	-17.6 ± 0.2
C32:2ω?	Sponge	-	-	0.4 ± 0.8	-22.0 ± 0.8	-	-

Supplementary References

1. Nieuwenhuize, J., Maas, Y. E. M. & Middelburg, J. J. Rapid analysis of organic carbon and nitrogen in particulate materials. *Mar. Chem.* **45**, 217–224 (1994).
2. Quinn, G. P. & Keough, M. J. *Experimental Design and Data Analysis for Biologists*. (Cambridge University Press, 2002). doi:DOI: 10.1017/CBO9780511806384
3. Jackson, A. L., Inger, R., Parnell, A. C. & Bearhop, S. Comparing isotopic niche widths among and within communities: SIBER - Stable Isotope Bayesian Ellipses in R. *J. Anim. Ecol.* **80**, 595–602 (2011).
4. Parnell, A. C., Inger, R., Bearhop, S. & Jackson, A. L. Source Partitioning Using Stable Isotopes: Coping with Too Much Variation. *PLoS One* **5**, e9672 (2010).
5. Griffith, D. R. *et al.* Carbon dynamics in the western Arctic Ocean: Insights from full-depth carbon isotope profiles of DIC, DOC, and POC. *Biogeosciences* **9**, 1217–1224 (2012).
6. Druffel, E. R. M., Griffin, S., Glynn, C. S., Benner, R. & Walker, B. D. Radiocarbon in dissolved organic and inorganic carbon of the Arctic Ocean. *Geophys. Res. Lett.* **44**, 2369–2376 (2017).
7. Thibodeau, B., Bauch, D. & Voss, M. Nitrogen dynamic in Eurasian coastal Arctic ecosystem: Insight from nitrogen isotope. *Global Biogeochem. Cycles* **31**, 836–849 (2017).
8. Benner, R., Louchouarn, P. & Amon, R. M. W. Terrigenous dissolved organic matter in the Arctic Ocean and its transport to surface and deep waters of the North Atlantic. *Global Biogeochem. Cycles* **19**, 1–11 (2005).
9. Kahn, A. S., Chu, J. W. F. & Leys, S. P. Trophic ecology of glass sponge reefs in the Strait of Georgia, British Columbia. *Sci. Rep.* **8**, 756 (2018).
10. Freeman, C. J. *et al.* Microbial symbionts and ecological divergence of Caribbean sponges: A new perspective on an ancient association. *ISME J.* **14**, 1571–1583 (2020).
11. Synal, H.-A., Stocker, M. & Suter, M. MICADAS: A new compact radiocarbon AMS system. *Nucl. Instruments Methods Phys. Res. Sect. B Beam Interact. with Mater. Atoms* **259**, 7–13 (2007).
12. Mollenhauer, G., Grotheer, H., Gentz, T., Bonk, E. & Hefter, J. Standard operation procedures and performance of the MICADAS radiocarbon laboratory at Alfred Wegener Institute (AWI), Germany. *Nucl. Instruments Methods Phys. Res. Sect. B Beam Interact. with Mater. Atoms* **496**, 45–51 (2021).
13. Wacker, L., Němec, M. & Bourquin, J. A revolutionary graphitisation system: Fully automated, compact and simple. *Nucl. Instruments Methods Phys. Res. Sect. B Beam Interact. with Mater. Atoms* **268**, 931–934 (2010).
14. Ruff, M. *et al.* Gaseous radiocarbon measurements of small samples. *Nucl. Instruments Methods Phys. Res. Sect. B Beam Interact. with Mater. Atoms* **268**, 790–794 (2010).
15. Stuiver, M. & Polach, H. A. Discussion Reporting of ^{14} C Data. *Radiocarbon* **19**, 355–363 (1977).
16. Winterfeld, M. *et al.* Deglacial mobilization of pre-aged terrestrial carbon from degrading permafrost. *Nat. Commun.* **9**, 3666 (2018).
17. Meyer, V. D. *et al.* Permafrost-carbon mobilization in Beringia caused by deglacial meltwater runoff, sea-level rise and warming. *Environ. Res. Lett.* **14**, 085003 (2019).
18. Wacker, L. *et al.* A versatile gas interface for routine radiocarbon analysis with a gas ion source. *Nucl. Instruments Methods Phys. Res. Sect. B Beam Interact. with Mater. Atoms* **294**, 315–319 (2013).
19. Muyzer, G., de Waal, E. C. & Uitterlinden, A. G. Profiling of complex microbial populations by denaturing gradient gel electrophoresis analysis of polymerase chain reaction-amplified genes coding for 16S rRNA. *Appl. Environ. Microbiol.* **59**, 695 LP – 700 (1993).
20. Caporaso, J. G. *et al.* Global patterns of 16S rRNA diversity at a depth of millions of sequences per sample. *Proc. Natl. Acad. Sci.* **108**, 4516 LP – 4522 (2011).

21. Bolyen, E. *et al.* Reproducible, interactive, scalable and extensible microbiome data science using QIIME 2. *Nat. Biotechnol.* **37**, 852–857 (2019).
22. Callahan, B. J. *et al.* DADA2: High-resolution sample inference from Illumina amplicon data. *Nat. Methods* **13**, 581–583 (2016).
