# Supplementary material for: Giant sponge grounds of Central Arctic seamounts are associated with extinct seep life
Source: Nat Commun. 2022 Feb 8;13:638. doi: 10.1038/s41467-022-28129-7 (PMC8826442; doi:10.1038/s41467-022-28129-7)
Supplement: Supplementary file 3 — Description of Additional Supplementary Files [file 41467_2022_28129_MOESM3_ESM.docx]

**Description of Additional Supplementary Files**

**Title: Supplementary Data 1.**

**Description:** *G. parva* metatranscriptomic expressed pathways and their percentage of completeness.

**Title: Supplementary Data 2.**

**Description:** Expression of bacterial and archaeal carbon fixation pathways in the *G. parva* metatranscriptome.

**Title: Supplementary Data 3.**

**Description:** Langseth Ridge sample list, locations, depths, gears and analyses performed for each samples. BC: box corer; DRG_C: dredge chain bag; ICE: Ice station; MUC: multi corer; MN: multi opening/closing net; NUI_ROV: Nereid under ice _ remotely operated vehicle.
