# Supplementary material for: Giant sponge grounds of Central Arctic seamounts are associated with extinct seep life
Source: Nat Commun. 2022 Feb 8;13:638. doi: 10.1038/s41467-022-28129-7 (PMC8826442; doi:10.1038/s41467-022-28129-7)
Supplement: Supplementary file 7 — Reporting Summary [file 41467_2022_28129_MOESM7_ESM.pdf]

nature portfolio

Corresponding author(s): Teresa Maria Morganti and Antje Boetius

Last updated by author(s): Dec 3, 2021

# Reporting Summary

Nature Portfolio wishes to improve the reproducibility of the work that we publish. This form provides structure for consistency and transparency in reporting. For further information on Nature Portfolio policies, see our Editorial Policies and the Editorial Policy Checklist.

## Statistics

For all statistical analyses, confirm that the following items are present in the figure legend, table legend, main text, or Methods section.

| n/a | Confirmed | |
|---|---|---|
| ☐ | ☒ | The exact sample size ($n$) for each experimental group/condition, given as a discrete number and unit of measurement |
| ☐ | ☒ | A statement on whether measurements were taken from distinct samples or whether the same sample was measured repeatedly |
| ☐ | ☒ | The statistical test(s) used AND whether they are one- or two-sided<br>*Only common tests should be described solely by name; describe more complex techniques in the Methods section.* |
| ☒ | ☐ | A description of all covariates tested |
| ☐ | ☒ | A description of any assumptions or corrections, such as tests of normality and adjustment for multiple comparisons |
| ☐ | ☒ | A full description of the statistical parameters including central tendency (e.g. means) or other basic estimates (e.g. regression coefficient) AND variation (e.g. standard deviation) or associated estimates of uncertainty (e.g. confidence intervals) |
| ☐ | ☒ | For null hypothesis testing, the test statistic (e.g. $F$, $t$, $r$) with confidence intervals, effect sizes, degrees of freedom and $P$ value noted<br>*Give P values as exact values whenever suitable.* |
| ☒ | ☐ | For Bayesian analysis, information on the choice of priors and Markov chain Monte Carlo settings |
| ☒ | ☐ | For hierarchical and complex designs, identification of the appropriate level for tests and full reporting of outcomes |
| ☒ | ☐ | Estimates of effect sizes (e.g. Cohen's $d$, Pearson's $r$), indicating how they were calculated |

*Our web collection on statistics for biologists contains articles on many of the points above.*

## Software and code

Policy information about availability of computer code

| | |
|---|---|
| Data collection | No specific software was designed and used to collect data |
| Data analysis | The software used for data analyses are: PAPARAZZI software application (Version 2.6) for sponge counting and size measuring, R studio (version 3.6.1) with the following packages lme4 v.1.1-21, SIBER v.2.1.4 [Stable Isotope Bayesian Ellipses] and Rlims used for the linear mixed models, isotopic niche area and lipids analyses, respectively.<br>DADA2 algorithm was used to exact Amplicon Sequence Variants (ASVs) and classified based on the Silva database.<br>The Linear Discriminant Analysis (LDA) Effect Size (LEfSe) algorithm was applied to identify significantly enriched microbial phyla in the host sponge.<br>FastQC was used to asses sequence quality of all read files. Trimmomatic v0.36 was used to trim metagenomic and metatranscriptomic reads, which were co-assembled with megahit v1.2.9 and Trinity v2.0.6, respectively. Small subunit (SSU) ribosomal genes were identified with the ssu_finder function of CheckM v1.0.12 and taxonomically annotated with the SINA. Coding regions were identified with TransDecoder v5.5.0 and PFAMs were annotated with Trinotate v3.2.0.<br>bowtie2 was used to quantify expression level. MEBS v1.2 was used for completeness estimations of various metabolic pathways. |

For manuscripts utilizing custom algorithms or software that are central to the research but not yet described in published literature, software must be made available to editors and reviewers. We strongly encourage code deposition in a community repository (e.g. GitHub). See the Nature Portfolio guidelines for submitting code & software for further information.

## Data

Policy information about availability of data

All manuscripts must include a data availability statement. This statement should provide the following information, where applicable:

- Accession codes, unique identifiers, or web links for publicly available datasets
- A description of any restrictions on data availability
- For clinical datasets or third party data, please ensure that the statement adheres to our policy

Source data are provided with this paper. The isotope data generated in this study are provided in the Supplementary Information and Source Data file. Seabed images used to estimate the sponge community biomass are publicly available from the PANGEA data archive (https://doi.pangaea.de/10.1594/PANGAEA.871550). The processed density and biomass data are available in OSF.IO database under the project Deep-Sea Arctic Geodia ground: sponge count and biomass (https://doi.org/10.17605/OSF.IO/VCXYE). Silva data used to classified amplicon sequences is available at https://www.arb-silva.de/. Metagenomic, metatranscriptomic and amplicon data are deposited in the NCBI database under BioProject PRJNA454581 (https://www.ncbi.nlm.nih.gov/bioproject/PRJNA454581) with accession code SRR7182305-17 for metatranscriptomic raw data and GIMA00000000 for metatranscriptomic co-assembly. The original cruise report is available at https://epic.awi.de/id/eprint/44286/ with the description and links to related published data from the field study.

# Field-specific reporting

Please select the one below that is the best fit for your research. If you are not sure, read the appropriate sections before making your selection.

☐ Life sciences ☐ Behavioural & social sciences ☒ Ecological, evolutionary & environmental sciences

For a reference copy of the document with all sections, see nature.com/documents/nr-reporting-summary-flat.pdf

# Ecological, evolutionary & environmental sciences study design

All studies must disclose on these points even when the disclosure is negative.

**Study description**

The analyses performed in this study are: video surveying, bulk stable isotope, PLFAs and compound specific isotope analysis, radiocarbon analysis, -omics, image analysis for population density and size estimates. Additional data come from oceanographic surveys and are cited in the cruise report

For bulk stable isotope analysis the sample location was considered as random factor in the linear mixed model. The number of replicates is listed in Table 1 and Supplementary Data 3. For each sponge individual from 2 to 9 replicates were analyzed.

The number of the analyzed individuals for PLFAs analysis were: Geodia parva n=9, Geodia hentscheli n=5, Stelletta rhaphidiophora n=3 as indicated in Supplementary Table 3. The number of replicates in the compound specific isotope analysis is indicated for each PLFAs in the box-plot Figure 4.

For -omics analysis only Geodia parva was considered, because it was the most abundant species. The number of sampled individuals was 13.

For the radiocarbon 14Δ analysis two Geodia parva sponge individuals were analyzed and 4 subsamples from different part of the sponge tissue were analyzed for the big individuals, while for the small individual were analyzed 2 subsamples.

The number of analyzed images was 696 distributed over the three summits (Karasik seamount, Central Mount and Northern mount) and central saddle.

**Research sample**

Megafauna, seawater and sediments from the summits of the Langseth Ridge, underwater seamount ridge from Gakkel Ridge, Central Arctic. These samples were collected in order to investigate different food sources used by sponge community.
The analyzed sponge species were: Geodia parva, G. henthsceli and Stelletta raphidiophora. This sponge population is representative of a typical Arctic-Boreal Geodia ground and were the most abundant species observed at the study site.

**Sampling strategy**

Sampling from the ship on random stations, repeated on 3 summits.
For sponge biomass and distribution, 4 transects were analyzed (89, 100, 120,169). For the first transect all images were analyzed and compared with every 10th analysis. No difference was found, therefore for the rest three transects it was analyzed one image every 10th.
For stable and compound specific isotope sample analyses no sample size calculation was performed beforehand. From two to nine replicates per sponge individual were performed to assess for internal variability. Such sample size provided low variance coefficient (<10%). For the calculation of the standard ellipse area (SEAc) only sample size >3 was used due to the high uncertainty in Bayesian standard ellipse in small sample size.
For omics analysis only G. parva species was analyzed because it was the most abundant species. A total of 11 G. parva specimens were used for amplicon and three seawater samples from each summits. While G. species specimens (n=13) were analyzed for metagenomic and metatranscriptomic analysis.
In order to have the same sampling method employed for omics analyses, only box-corer sampled species were used

| | |
|---|---|
| Data collection | The ship sonars were used for mapping, and seafloor images were taken using Ocean Floor Observation and Bathymetry System (OFOBS).<br>Sponge samples, together with spicule-tube mat, sediment and associated macrofauna were collected via camera-guided multiple corer (TV-MUC), box-corer (0.25 m2), dredge with chain bag and "Nereid Under Ice" remotely operated vehicle (NUI ROV), and seawater samples were obtained from CTD-Rosette casts.<br>Zooplankton and fecal pellets of Oikopleura sp. were collected using a multinet equipped with nine nets (150 µm mesh size). Several casts were carried out on top of the three mounts collecting plankton from a range of sampling depth intervals (500-400-300-200-100-50-25-0 m). Particulate organic matter (POM) was collected from 10 m depth using CTD-rosette and filtered through Whatmann GF/F filters (0.7µm, 10-14 liters per filter).<br><br>The responsibles of the sampling collection were: Beate Slaby, Jennifer Dannheim and Autun Purser. The chief scientist of the expedition was Antje Boetius who planned the experiments and surveys. |
| Timing and spatial scale | The samples were collected during the interdisciplinary research expedition PS101 from the 9th of September to the 23rd of October 2016. In the specific, the samples used for this study were collected from 18/09/2016 to 8/10/2016. The sampling date and location (lat. and long.) are listed in Supplementary Data 3 excel file. |
| Data exclusions | In the radiocarbon dating #14C analysis one subsample from the large sponge specimen was found to contain 14C contamination (F14C value >> 1) from an unknown source, and therefore was excluded from further analysis |
| Reproducibility | The study is a field study and hence reproducible. This study does not involve any manipulative laboratory experiments. |
| Randomization | The surveys and station selection was randomly distributed over the different summits. Sponge samples, sediment, ambient water, organic debris were collected over the three summits (Norther mount, Karasik semounts and Central Mounts) and the saddle (Central Mount Saddle). Bulk sponge isotopic values (δ13C and δ15N) were analyzed using a linear mixed model effect with sponge species as the fixed factor (level 4: G. parva, G. hentscheli, S. rhaphidiophora and G. parva buds) and sampling sites (the summits and the central mount saddle) as the random factor in order to test for differences between sponge species controlled by the variability of the random factor. Sample location did not show any significant difference and therefore the samples were analyzed together. |
| Blinding | Blinding is not relevant to our study |

Did the study involve field work? ☒ Yes ☐ No

## Field work, collection and transport

| | |
|---|---|
| Field conditions | During the PSP101 the winds ranged from 1 to 11 Bft with most direction toward South, and weather temperature rose near zero up to dropping below -10°C. Sea ice was present throughout the study |
| Location | The main sampling location was Gakkel Ridge, extending from 87°N 62°E to 85°55'N 57°45'E for approximately 125 km in the central Arctic Ocean. This location is in the high seas, and not part of the EEZ |
| Access & import/export | Gakkel Ridge is situated in international water where no sampling permit is needed.<br>The PS101 expedition began on 9 September leaving the port of Tromso. After several stops in for deployment of oceanographic buoys in sites of interest such as in Norwegian waters, on September 11 the ice edge was reached at 81.5°N. On the18th of September the cruise reached the Karasik seamounts and the field-work/sampling collection at the Gakkel Ridge lasted up to the 11th of October. In the following days other sampling locations of interest, suchs as FRAM strait, were covered by the research cruise PS101 and the transit back to Bremerhaven ended the 23rd of October. The import of samples was done according to the relevant rules (no special permit required) |
| Disturbance | A possible disturbance caused by this study is the effect of sampling on deep sea life, however, as samples were immediately treated, i.e. fixed or frozen, it is deemed negligible for this study. |

# Reporting for specific materials, systems and methods

We require information from authors about some types of materials, experimental systems and methods used in many studies. Here, indicate whether each material, system or method listed is relevant to your study. If you are not sure if a list item applies to your research, read the appropriate section before selecting a response.

## Materials & experimental systems

| n/a | Involved in the study |
| --- | --- |
| ☒ | ☐ Antibodies |
| ☒ | ☐ Eukaryotic cell lines |
| ☒ | ☐ Palaeontology and archaeology |
| ☐ | ☒ Animals and other organisms |
| ☒ | ☐ Human research participants |
| ☒ | ☐ Clinical data |
| ☒ | ☐ Dual use research of concern |

## Methods

| n/a | Involved in the study |
| --- | --- |
| ☒ | ☐ ChIP-seq |
| ☒ | ☐ Flow cytometry |
| ☒ | ☐ MRI-based neuroimaging |

# Animals and other organisms

Policy information about studies involving animals; ARRIVE guidelines recommended for reporting animal research

| | |
| --- | --- |
| Laboratory animals | Np laboratory animals were used. |
| Wild animals | The study sponge population is characterized by dense and massive sponge individuals living on top of spicule mat intermixed with organic detritus from past seep community. The most abundant species were Geodia hentscheli, Geodia parva and Stelletta rhaphidiophora. Abundant juveniles and buds were also observed. The sponges were collected via camera-guided multiple corer (TV-MUC), box-corer (0.25 m2), dredge with chain bag and "Nereid Under Ice" remotely operated vehicle (NUI ROV).<br>After the collection the sponges were sorted and frozen at -20°C and -80°C depending on the analysis.<br>No experiments with live animals were performed. |
| Field-collected samples | The samples were collected in the field and no experiments with live animals were performed. |
| Ethics oversight | No ethical approval or guidance was required as the study animals are invertebrates |

Note that full information on the approval of the study protocol must also be provided in the manuscript.
